# Supplementary material for: Understanding Salinity-Driven Modulation of Microbial Interactions: Rhizosphere versus Edaphic Microbiome Dynamics
Source: Microorganisms. 2024 Mar 28;12(4):683. doi: 10.3390/microorganisms12040683 (PMC11052110; doi:10.3390/microorganisms12040683)
Supplement: Supplementary file 1 [file microorganisms-12-00683-s001.zip › microorganisms-2922606-supplementary.pdf]

Supplementary material for

Understanding Salinity-Driven Modulation of Microbial Interactions: Rhizosphere Versus Edaphic Microbiome Dynamics

Rui Li [1,2], Haihua Jiao [1,3], Bo Sun [1,2], Manjiao Song [1,2], Gaojun Yan [1,2], Zhihui Bai [1,2], Jiancheng Wang [4,*], Xuliang Zhuang [1,2,5,], Qing Hu [1,2,6,*]

1 Research Center for Eco-Environmental Sciences, Chinese Academy of Sciences, Beijing 100085, China
2 University of Chinese Academy of Sciences, Beijing 100049, China
3 Department of Biological Sciences and Technology, Changzhi University, Changzhi, 046011, China
4 Binzhou Institute of Technology, Weiqiao-UCAS Science and Technology Park, Binzhou, Shandong 256606, China
5 Institute of Tibetan Plateau Research, Chinese Academy of Sciences, Beijing 100101, China
6 Xiongan Innovation Institute, Xiongan New Area, Hebei 071000, China
* Correspondence: huqing@xii.ac.cn (Q.H.), wangjiancheng@wqucas.com (J. W.)

Table S1. Characteristics of different sample groups based on salinity level and soil environment.

Group	Electrical conductivity (ds/m)	Soil environment
R1	<2	Rhizosphere
R2	2–4	Rhizosphere
R3	4–8	Rhizosphere
R4	>8	Rhizosphere
N1	<8	Non-rhizosphere
N2	8–12	Non-rhizosphere
N3	12–14	Non-rhizosphere
N4	>14	Non-rhizosphere

Table S2. Topological properties of bipartite fungal–bacterial networks in non-rhizosphere and rhizosphere soils.

Topological property	Non-rhizosphere	Rhizosphere
number.of.species.Fungus	37.000	31.000
number.of.species.Bacteria	286.000	204.000
mean.number.of.links.Fungus	27.027	48.290
mean.number.of.links.Bacteria	3.497	7.338
mean.number.of.shared.partners.Fungus	3.266	17.622
mean.number.of.shared.partners.Bacteria	0.573	2.088
cluster.coefficient.Fungus	0.095	0.237
cluster.coefficient.Bacteria	0.095	0.237
weighted.cluster.coefficient.Fungus	0.535	0.868
weighted.cluster.coefficient.Bacteria	0.908	0.996
niche.overlap.Fungus	0.088	0.319
niche.overlap.Bacteria	0.126	0.211
togetherness.Fungus	3.650E-02	1.970E-01
togetherness.Bacteria	0.057	0.117

C.score.Fungus	0.793	0.443
C.score.Bacteria	0.750	0.572
V.ratio.Fungus	24.186	17.047
V.ratio.Bacteria	2	6
discrepancy.Fungus	619	654
discrepancy.Bacteria	625.000	613.000
extinction.slope.Fungus	2.589	4.764
extinction.slope.Bacteria	15.801	43.757
robustness.Fungus	0.713	0.806
robustness.Bacteria	0.922	0.973
functional.complementarity.Fungus	200.460	196.122
functional.complementarity.Bacteria	346.180	364.927
partner.diversity.Fungus	2.877	3.733
partner.diversity.Bacteria	0.981	1.616
generality.Fungus	27.027	48.290
vulnerability.Bacteria	3.497	7.338

Table S3. Topological properties of bacterial co-occurrence networks in non-rhizosphere and rhizosphere soils.

Topological property	Non-rhizosphere				Rhizosphere			
	N1	N2	N3	N4	R1	R2	R3	R4
Total nodes	779	560	460	269	103	243	65	206
Total links	2588	1932	1017	1537	194	2466	72	680
R square of power-law	0.970	0.976	0.979	0.979	0.976	0.971	0.997	0.984
Average degree (avgK)	6.644	6.900	4.422	11.428	3.767	20.296	2.215	6.602
Average clustering coefficient (avgCC)	0.209	0.232	0.222	0.407	0.333	0.573	0.156	0.375
Average path distance (GD)	4.619	4.837	5.043	3.562	3.469	2.561	5.066	3.691
Geodesic efficiency (E)	0.244	0.245	0.227	0.346	0.348	0.451	0.287	0.328
Harmonic geodesic distance (HD)	4.095	4.081	4.399	2.893	2.873	2.217	3.480	3.046
Maximal degree	46	54	31	69	21	108	11	45
Centralization of degree (CD)	0.051	0.084	0.058	0.215	0.169	0.362	0.137	0.187
Maximal betweenness	15957	9273	14008	3991	1194	5495	647	3939
Centralization of betweenness (CB)	0.048	0.054	0.125	0.103	0.217	0.183	0.284	0.177
Maximal stress centrality	2.39E+05	1.31E+05	9.44E+04	6.41E+04	3.07E+03	4.98E+04	8.93E+02	2.52E+04
Centralization of stress centrality (CS)	0.742	0.773	0.846	1.623	0.551	1.627	0.393	1.121
Maximal eigenvector centrality	1	1	1	1	1	1	1	1
Centralization of eigenvector centrality (CE)	0.950	0.932	0.948	0.837	0.863	0.786	0.888	0.878
Maximal closeness centrality	1.14E-04	4.68E-05	1.86E-04	3.05E-04	4.89E-04	4.31E-04	9.79E-04	7.61E-04
Centralization of closeness centrality (CCL)	0.020	0.005	0.019	0.020	0.018	0.024	0.027	0.053
Density (D)	0.009	0.012	0.010	0.043	0.037	0.084	0.035	0.032
Reciprocity	1	1	1	1	1	1	1	1
Transitivity (Trans)	0.253	0.377	0.213	0.430	0.277	0.495	0.131	0.315
Connectedness (Con)	0.980	0.879	0.966	0.927	0.685	0.935	0.641	0.962
Efficiency	0.993	0.988	0.992	0.958	0.959	0.914	0.967	0.971
Hierarchy	0	0	0	0	0	0	0	0

Lubness	1	1	1	1	1	1	1	1
module	19	25	13	10	14	11	14	11
modularity	0.500	0.571	0.702	0.894	0.587	0.287	0.688	0.486
vulnerability	0.018	0.022	0.036	0.043	0.160	0.041	0.295	0.055

The different salinity levels in non-rhizosphere soil were classified as N1-N4, while the different salinity levels in rhizosphere soil were classified as R1-R4. The specific criteria for this classification were shown in Table S1.

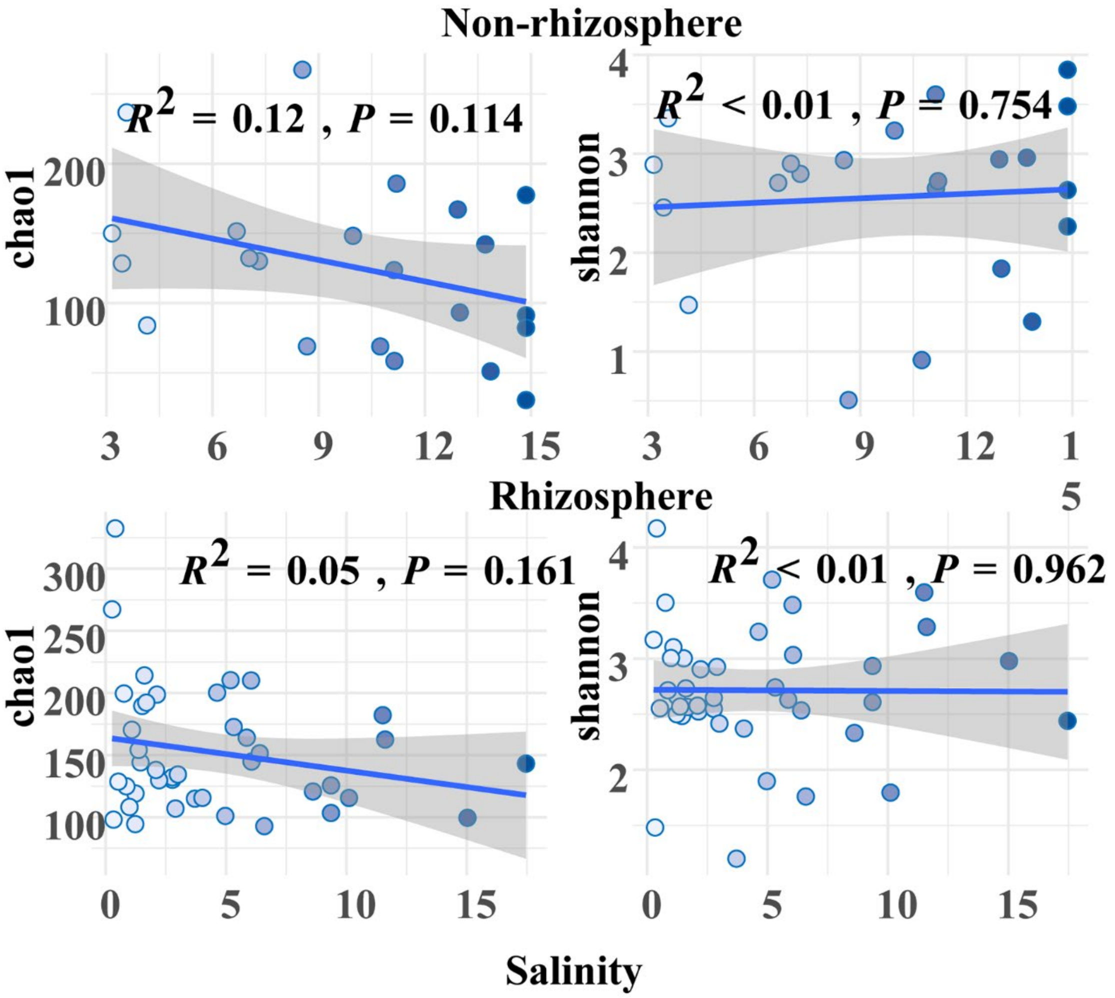

Figure S1. Linear regression analysis of fungal alpha diversity against salinity in non-rhizosphere and rhizosphere soils.

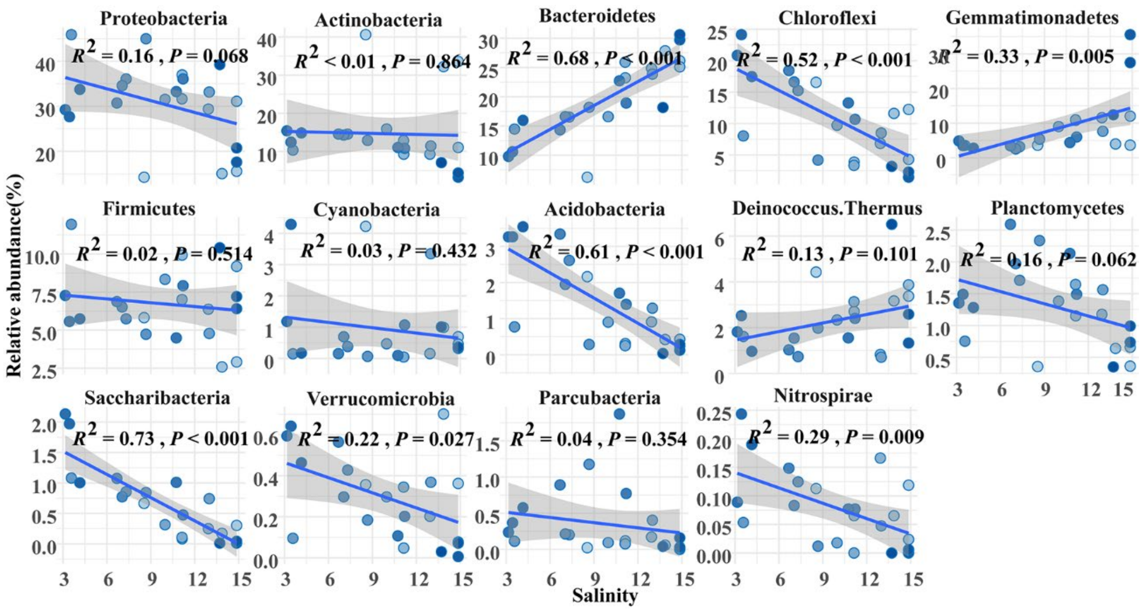

Figure S2. Linear regression analysis of the relative abundance of non-rhizosphere bacterial taxa (phylum) with against salinity in non-rhizosphere soil.

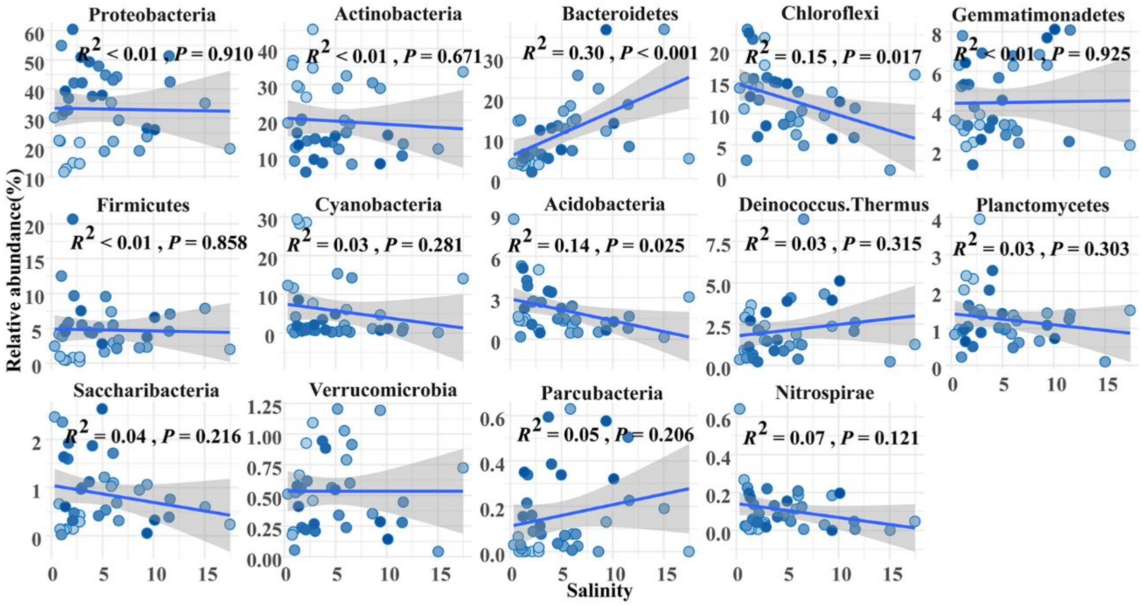

Figure S3. Linear regression analysis of the relative abundance of rhizosphere bacterial taxa (phylum level) against salinity.

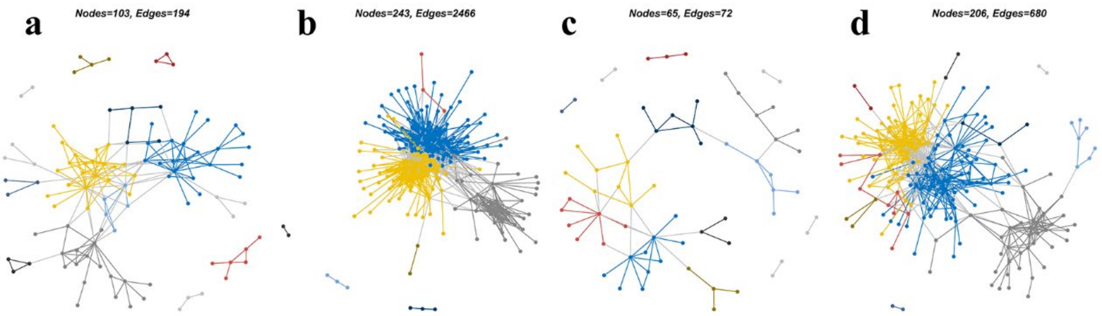

Figure S4. Overview of the evolution of bacterial ecological network structure in rhizosphere soil under different salinity levels (R1: c, R2: d, R3: e, R4: f). The nodes and connections are colored according to module attributes. The largest module is colored differently and the remaining smaller modules are colored gray. The different salinity levels in rhizosphere soil were classified as R1-R4. The specific criteria for this classification were shown in Table S1.
